# Population Parameters and Feeding Preference of *Spodoptera litura* (Lepidoptera: Noctuidae) on Different *Asparagus officinalis* Tissues

**DOI:** 10.3390/insects13121149

**Published:** 2022-12-13

**Authors:** Li-Min Ye, Xue-Yuan Di, Bin Yan, Jian-Feng Liu, Xiu-Qin Wang, Mao-Fa Yang

**Affiliations:** 1 Guizhou Provincial Key Laboratory for Agricultural Pest Management of the Mountainous Region, Institute of Entomology, Scientific Observing and Experimental Station of Crop Pest in Guiyang, Ministry of Agriculture, Guizhou University, Guiyang 550025, China; 2 College of Tobacco Science, Guizhou University, Guiyang 550025, China

**Keywords:** *Spodoptera litura*, *Asparagus officinalis*, life table, feeding preference

## Abstract

**Simple Summary:**

The population parameters and feeding preference of *Spodoptera litura* on asparagus are unclear. We studied the population parameters and feeding preference of *S. litura* on asparagus stems and leaves. The results showed that *S. litura* could complete growth and development on both stems and leaves of asparagus. The developmental duration of larvae on stems was significantly shorter than that on leaves. The survival rate, the intrinsic rate of increase, and the finite rate of increase of *S. litura* fed on stems were higher than those fed on leaves. Therefore, asparagus stems were more suitable for its population growth. However, *S. litura* larvae (except for the 6th instar at 2.5 h) did not prefer to choose stems that were more conducive to their population growth. The 3rd and 5th instar larvae preferred asparagus leaves. There was no significant difference in feeding preference on stems and leaves at the age of 1, 2, 4, and 6 (except 2.5 h). Thus, we found that this pest was harmful both on asparagus stems and leaves. In the field, we will focus on stems and leaves in early prevention of this pest.

**Abstract:**

*Spodoptera litura* is an important pest that seriously affects *Asparagus officinalis* production. To clarify the population characteristics and feeding preference of *S. litura* on different asparagus tissues, asparagus stems and leaves were selected as the research objects, related studies were conducted by constructing the life table and the feeding preference experiment. The results showed that *S. litura* could complete its development and reproduction normally on asparagus stems or leaves. Although the adult longevity and fecundity of *S. litura* on the two types of tissues were not significantly different, the development duration of larvae and pupae, and total preoviposition period on leaves were significantly longer than those raised on stems. The intrinsic rate of increase and finite rate of increase were 0.186 d^−1^ and 1.204 d^−1^ on stems, which were significantly higher than those fed on leaves (0.161 d^−1^ and 1.175 d^−1^). The mean generation time on stems (32.88 d) was significantly lower than on leaves (36.88 d). It indicated that stems were more suitable for its population growth. In the feeding preference, the third and fifth instar larvae preferred to feed on leaves, and other instar larvae (except for the sixth instar of 2.5 h) had no significant difference. These results will provide a theoretical reference for further research and forecasting and integrated control.

## 1. Introduction

*Spodoptera litura* is a worldwide distributed polyphagous agricultural pest belonging to Lepidoptera, Noctuidae, which can harm more than 300 kinds of plants [1,2]. Asparagus is one of its host plants and an economically important crop. *Asparagus officinalis* L. is an herbaceous perennial plant belonging to the genus *Asparagus* of the family Liliaceae. The fleshy stem is the edible part of asparagus; it can be marketed as a fresh vegetable or processed into canned food to meet the growing market demand [3,4,5]. The leaves are the main photosynthetic organs of asparagus, which can produce nutrients for the formation of young asparagus stems [6,7,8]. *Spodoptera litura* is one of the main pests of asparagus, and its damage to asparagus has been increasing in recent years [9]. *Spodoptera litura* larvae cluster and gnaw on the leaves and branches, resulting in defoliation and reducing its photosynthesis and carbohydrate assimilation capacity, causing the asparagus to bend and collapse, resulting in the death of the asparagus plant [10]. *Spodoptera litura* has a strong reproductive ability, strong ecological adaptation, severe generation overlap, and irregular outbreaks, which pose a serious threat to the safe production of asparagus [11]. Numerous studies have demonstrated that different hosts that insects feed on have effects on their growth and development, fecundity, weight, and nutritional level [12,13]. Similarly, feeding on different parts of hosts also affects insect feeding behavior, viability, and fecundity [14,15,16]. For example, different maize tissues significantly affected the growth, development, and fecundity of *Spodoptera frugiperda* [17]. The larvae of *Helicoverpa zea* had different survival rates on different tissue types of soybeans and preferentially fed on various tissue types during different larval development stages [18]. Different parts of wheat seedlings had different impacts on the production period and weight of *Rhopalosiphum padi* [19]. Furthermore, Tu [20] reported that the larvae of *Brithys crini* mostly preferred to feed on the tender floral organs rather than the dense corm of *Zephyranthes candida*. At present, there have been a large number of reports about the growth, development, and feeding preference of *S. litura* on different hosts [21,22,23], but there have been few studies about the effect of different parts of plant hosts on the performance of *S. litura*. For example, Hu et al. [16] used soybean organs at different reproductive stages to evaluate their resistance against *S. litura*. Wu [15] explored the host selectivity of *S. litura* on different growth periods of cabbage and soybeans. Yue et al. [24] studied the effects of *S. litura* feeding on the defensive enzyme activities in leaves at different parts of kidney bean plants. At the same time, there were also many reports on the growth and development of insects on asparagus [25,26,27,28], but the research on *S. litura* to asparagus focused on its occurrence dynamics and control. So far, only Wang et al [29] have studied the growth, development, and reproduction of *S. litura* on asparagus. However, the growth and feeding preference of *S. litura* on different tissues of asparagus have not been reported. A life table is an important tool to study the effects of external factors on insect development, reproduction, survival, and population dynamics [30,31]. However, the traditional life table has some limitations: it only describes the role of females in the population and ignores the influence of male individual development and age changes on the population, so it cannot accurately describe the changes in the entire insect population [32]. In contrast, the age-stage, two-sex life table considers stage differentiation and both sexes precisely, which can describe the development, survival rate, reproduction, and population growth more comprehensively and accurately [33,34]. At present, the age-stage, two-sex life table has been widely used in the study of *Bactrocera cucurbitae* [35], *Hippodamia variegata* [36], *Conogethes punctiferalis* [37], *S. frugiperda*, [38], etc. The age-stage, two-sex life table has also been used in research on *S*. *litura* [21,39,40]. Except for life table data, the feeding preference behavior of insects is also one of the indicators to evaluate the fitness of insects to hosts [41]. Host selection behavior of herbivorous insects is extremely important for their behavioral adaptation and survival [42,43]. Asparagus stems and leaves are the two most important parts of asparagus. However, the development, reproduction, and feeding preferences of *S. litura* on stems and leaves are still unclear. If we understand the growth, development, reproduction, and feeding preference of *S. litura* on stems and leaves, it will help us carry out targeted prevention and control of *S. litura*. Therefore, in this study, we collected life table data and constructed a life table to measure the growth and development of *S. litura* feeding on asparagus stems and leaves, and we determined the feeding preference of *S. litura* on asparagus tissues.

## 2. Materials and Methods

### 2.1. Insects

*Spodoptera litura* larvae were collected in July 2021 from an asparagus field in Xinmeng village, Mochong Town, Duyun City, Guizhou Province (25°55′53″ N, 107°55′14″ E), and reared on asparagus stems and leaves for more than 3 consecutive generations in a laboratory. All the *S. litura* individuals were kept at 27 ± 1 °C, under 75 ± 5% relative humidity, and with a photoperiod of 14 L:10 D [44].

The larvae were fed with fresh asparagus stems and leaves every day. The pupae at 4–5 days of age were sexed and kept in separate plastic containers. The newly emerged adults were transferred into an oviposition container, lined with wax paper, and provided with a cotton ball dipped in 10% honey solution. The eggs were collected daily and put in the culture dishes in an illumination incubator under the above conditions. The newly hatched larvae were put in a fresh host culture container.

### 2.2. Plants

Asparagus was provided by the asparagus planting base in Mochong Town, Duyun City, Guizhou Province, and planted in the experimental field of Institute of Entomology of Guizhou University.

### 2.3. Measurement of Life History Traits

Ten pairs of newly emerged adults of *S. litura* were randomly selected from the mass-rearing colony kept on either asparagus stems or leaves and placed in a plastic cup (bottom diameter: 5.7 cm, top diameter: 9.5 cm, height: 14.8 cm). They were provided with 10% honey solution on a cotton ball. One-hundred eggs laid on the same day were collected and placed in a Petri dish (diameter: 9 cm, height: 2 cm) lined with moist filter paper and covered with plastic wrap. The plastic wrap was pierced with 10 to 20 small holes with a diameter of 1 mm. The hatching of eggs was observed and calculated daily. Hatched larvae were transferred and reared individually in a plastic cup (bottom diameter: 3 cm, top diameter: 4 cm, height: 4 cm) and provided with either asparagus stems or leaves. The host plants were replaced and the feces were removed every day. When the larvae developed to 5th instar larvae, sterilized peat soil (2 cm in depth) was added into these test containers for pupation. Individual larvae were observed daily for molting and mortality [22,45]. On the first day of adult emergence, the male and female adults fed on the same host were paired up one by one and placed in a plastic cup (bottom diameter: 5.7 cm, top diameter: 9.5 cm, height: 14.8 cm). The top of the plastic cup was covered with 160 mesh gauze, and the circular wall was lined with paraffin wax papers. The adults were provided with fresh 10% honey solution to supplement their nutrients. If there were insufficient individuals of one sex emerging at the same time, another adult of the opposite sex was supplied from the mass-rearing colony kept on either asparagus stems or leaves for mating. If a female died before the male, a new female was added from the respective mass-reared population for mating purposes, but only the longevity of the male was recorded. If the male died before the female, we added a new male but only the longevity and fecundity of the female were recorded [46]. The number of eggs, oviposition days, and longevity of each individual were recorded daily until all individuals died.

### 2.4. Determination of Life Table Parameters

The raw data of *S. litura* life parameters were calculated and analyzed based on the age-stage, two-sex life table theory [47,48]. The population parameters were calculated and included the age-stage-specific survival rate (*s_xj_*), age-stage-specific fecundity (*f_xj_*), age-specific survival rates (*l_x_*), age-specific fecundity (*m_x_*), age-specific net maternity (*l_x_m_x_*), age-stage-specific life expectancy (*e_xj_*), and age-stage-specific reproductive value (*v_xj_*), as well as the intrinsic rate of increase (*r*), finite rate of increase *(λ)*, net reproductive rate (*R_0_*), and mean generation time (*T*).

*S_xj_* is the survival probability of *S. litura* from egg to *x* days old at the developmental stage *j*; *f_xj_* is the age-specific fecundity at age *x* and developmental stage *j*; *l_x_* is the survival probability of *S. litura* from egg to *x* days old; *m_x_* is the average population fecundity from egg to x days old; *l_x_m_x_* is the net fecundity of the population at age *x*. The age-stage-specific life expectancy (*e_xj_*) estimates the time that an individual at age *x* and stage *j* is expected to live [49]. The age-stage-specific reproductive value (*v_xj_*) predicts the contribution of an individual from age *x* and stage *j* to the future population. *r* refers to the maximum instantaneous growth rate of a population with a stable age-matched population under given physical and biological conditions; *λ* is the average daily growth rate of the population without the restriction of the external environment; *R_0_* refers to the total number of progeny that a population can reproduce in a generation under certain conditions; *T* is the time required for the pest population to increase to *R_0_*-fold of its population size at the stable stage distribution [50].

All calculation formulas are as follows:

(1) $$l_{x}=\sum_{j=1}^{m}s_{xj},$$

(2) $$m_{x}=\frac{\sum_{j=1}^{m}s_{xj}f_{xj}}{\sum_{j=1}^{m}s_{xj}},$$

(3) $$R_{0}=\sum_{x=0}^{\infty }l_{x}m_{x},$$

(4) $$\sum_{x=0}^{\infty }e^{-r(x+1)}l_{x}m_{x}=1,$$

(5) $$T=\frac{\ln R_{0}}{r},$$

(6) $$e_{xj}=\sum_{i=x}^{\infty }\sum_{y=j}^{m}s_{iy}^{\prime },$$

(7) $$v_{xj}=\frac{e^{r(x+1)}}{s_{xj}}\sum_{i=x}^{\infty }e^{-r(i+1)}\sum_{y=j}^{m}s_{iy}^{\prime }f_{iy},$$

### 2.5. Population Projection

The population growth and stage structure of *S. litura* on asparagus stems and leaves with an initial population of 10 eggs for the next 60 days were projected by incorporating the age-stage, two-sex life table data into the TIMING-MSChart program (TIMING-MSChart is available at http://140.120.197.173/Ecology/prod02.htm, accessed on 13 November 2022) [51].

### 2.6. Feeding Preference

Before the feeding preference experiment, an artificial diet was used to feed the larvae. The artificial diet was prepared by mixing the following materials: adding 400 mL distilled water, 16 g agar powder, 80 g soybean meal, 32 g yeast extract, 80 g bran, and 16 g casein and boiling for 30 min; then, 1.6 g sorbic acid was added to cool the solution to 60 °C. Then, adding 0.8 g choline chloride, 0.16 g cholesterol, 6.4 g vitamin C, and 0.16 g inositol, the solution was cooled and stored at 4 °C [40].

The 1st to 3rd instar larvae were placed in 15 cm glass Petri dishes, and the 4th to 6th instar larvae were placed in 20 cm glass Petri dishes. All the larvae used in the experiment were starved for 24 h in advance. The asparagus stems were placed on the left side, and the asparagus leaves were placed on the right side. Each instar larva of *S. litura* was placed in the central blank of the glass Petri dishes.

Ten larvae were placed in each treatment. The number of *S. litura* larvae on asparagus stems and leaves was recorded for 0.5 h, 1 h, 1.5 h, 2 h, 2.5 h, and 3 h, respectively [52]. Each experimental treatment was replicated 10 times in conditions of 27 ± 1 °C, 75 ± 5% (R. H), and 14 L:10 D in the RXZ-380A illumination incubators.

### 2.7. Data analysis

One-way analysis of variance (ANOVA) was performed using SPSS 26.0 to determine the differences in different treatment levels. Percentage data were subjected to arcsine square root transformation prior to ANOVA. Tukey’s honestly significant difference (HSD) was used as a post hoc test [53]. Population life table data were analyzed by the TWOSEX-MSChart program (TWOSEX-MSChart is available at http://140.120.197.173/Ecology/ accessed on 6 January 2021) [54]. The standard errors of each parameter were estimated by the bootstrap technique, with 100,000 samples; the paired bootstrap test was applied to test the differences among evaluated parameters [55]; all the graphs were created using SigmaPlot Version 14.0.

## 3. Results

### 3.1. Development Time and Reproductive Parameters

The developmental time, longevity, and reproductive parameters of *S. litura* fed on asparagus stems and leaves are presented in Table 1 and Table 2. Except for the egg and prepupa, the developmental time of the 1st to 6th instar larvae, preadult, and pupal stage of *S. litura* reared on asparagus stems were significantly shorter than those reared on asparagus leaves (*p* < 0.05) (Table 1). The total preoviposition period (TPOP) of *S. litura* fed on asparagus stems was 32.00 d, significantly shorter than the 36.56 d of *S. litura* fed on asparagus leaves (*p* < 0.05) (Table 2). There was no significant difference between stem-feeding *S. litura* and leaf-feeding *S. litura* in their adult preoviposition period (APOP), adult longevity, fecundity, and oviposition days (*p* > 0.05) (Table 2).

### 3.2. Survival Rate and Fecundity

**Figure 1:**
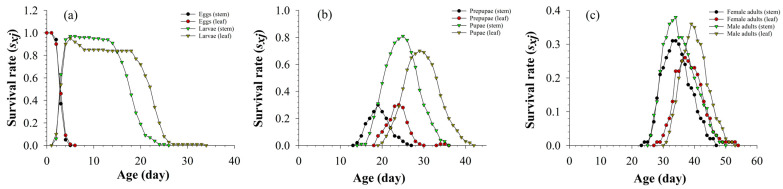
Survival rate of eggs and larvae (**a**), prepupae and pupae (**b**), female adults and male adults (**c**) of *S. litura* fed on different tissues of asparagus.

The age-stage-specific survival rate (*s_xj_*) of *S. litura* is shown in Figure 1. As can be seen from the picture, the survival rate of eggs, prepupae, and males was not affected by the two tissues. The survival rate of larvae on stems and leaves reached the maximum value on the 5th day, and the survival rate of larvae on stems began to decrease on the 13th day. This indicated that the larvae on the stems had partially entered the pupation state around the 13th day, which reduced the survival rate of larvae. The survival rate of larvae on stems was higher than that on leaves at the early stage of 13 days. The larvae survival rate on leaves began to decline at 18 days. Maximum survival rates of pupae and females on stems are higher than those on leaves. The survival rates of male adults of *S. litura* fed on both asparagus stems and leaves were higher than those of female adults (Figure 1).

**Figure 2:**
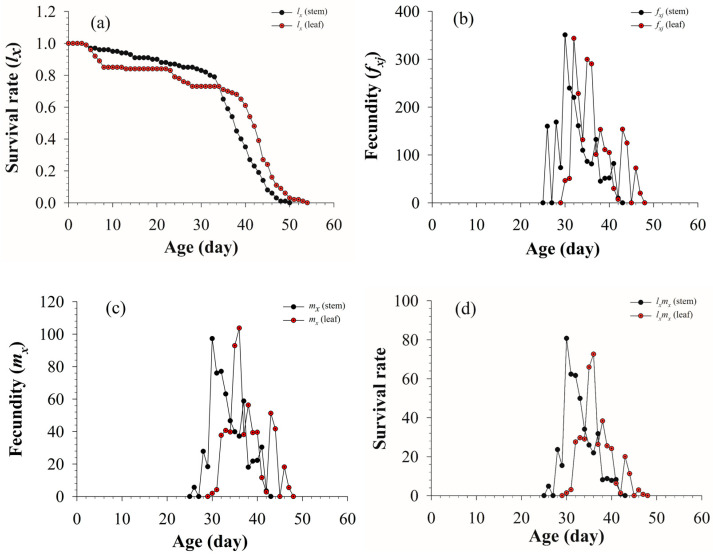
Population survival rate (*l_x_*) (**a**), fecundity of female adult (*f_xj_*) (**b**), population fecundity (*m_x_*) (**c**), and population maternity (*l_x_m_x_*) (**d**) of *S. litura* fed on different tissues of asparagus.

The age-specific survival rate (*l_x_*), female age-specific fecundity (*f_x__j_*), age-specific fecundity (*m_x_*), and age-specific net maternity (*l_x_m_x_*) for *S. litura* fed on asparagus stems and leaves are shown in Figure 2. The *l*_x_ curve of *S. litura* fed on stems was significantly decreased from age 33 d, and its survival rate decreased to zero by day 50, whereas the age-specific survival rate of *S. litura* fed on asparagus leaves gradually decreased from day 4, its survival rate dropped rapidly from day 38, and by day 54, it had dropped to zero. With the extension of time, the *f_x__j_*, *m_x_*, and *l_x_m_x_* curves increased first and then decreased, and the fecundity curves of *S. litura* fed on asparagus stems (*f_x__j_*, *m_x_*, and *l_x_m_x_*) began on the 25th day, whilst that of *S. litura* fed on asparagus leaves began on day 29. The two tissues had little effect on *f_x__j_*, and *m_x_*, the maximum value of *l_x_m_x_* on stems (80.72) was higher than that on leaves (72.61) (Figure 2).

### 3.3. Life Expectancy

**Figure 3:**
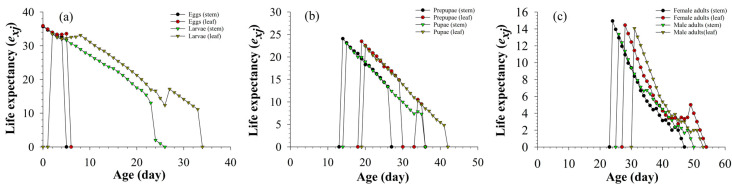
Age-stage-specific life expectancy (*e_xj_*) of eggs and larvae (**a**), prepupae and pupae (**b**), female adults and male adults (**c**) of *S. litura* fed on different tissues of asparagus.

Figure 3 shows that the age-stage-specific life expectancy (*e_xj_*) recorded under all of the treatments decreased with time. Two tissues did not affect pupal, prepupal, female, and male life expectancy; the life expectancy of larvae fed on stems was lower than that of larvae fed on leaves.

### 3.4. Reproductive Value

**Figure 4:**
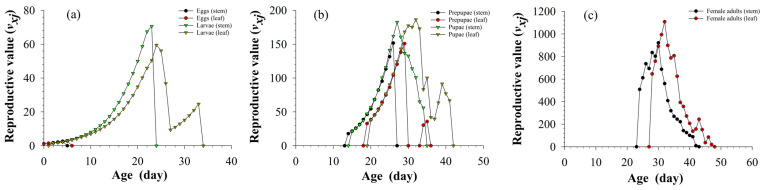
Age-stage-specific reproductive value (*v_xj_*) of eggs and larvae (**a**), prepupae and pupae (**b**), female adults and male adults (**c**) of *S. litura* fed on different tissues of asparagus.

It can be seen from the reproduction values in Figure 4 that stems and leaves had little effect on the reproduction values of eggs, prepupae, and pupae. The duration of the reproduction value of larvae on stems was 24 days, and the maximum reproduction value was 70.60. The duration of the reproduction value of larvae on leaves was 34 days, and the maximum reproduction value was 59.53. In the female adult period, the reproduction values reached the maximum, the reproduction value of *S. litura* fed on asparagus leaves reached the maximum value of 1108.20 on day 32, later than that of *S. litura* fed on asparagus stems, and its maximum value was higher than that fed on stems (the reproduction value reached the maximum value of 921.58 on the day 30).

### 3.5. Population Parameters

The intrinsic rate of increase (*r*) and the finite rate of increase (*λ*) of the *S. litura* population fed on asparagus stems were 0.186 d^−1^ and 1.204 d^−1^, respectively, which were significantly higher than those fed on asparagus leaves (0.161 d^−1^ and 1.175 d^−1^, *p* < 0.05). The net reproductive rate (*R_0_*) of *S. litura* fed on asparagus stems was 445.65, which was not significantly higher than that of those fed on asparagus leaves (385.75, *p* > 0.05). The mean generation time (*T*) of *S. litura* fed on asparagus stems was 32.88 d, which was significantly lower than that of those fed on leaves (36.88 d, *p* < 0.05, Table 3).

### 3.6. Population Dynamic Simulation

**Figure 5:**
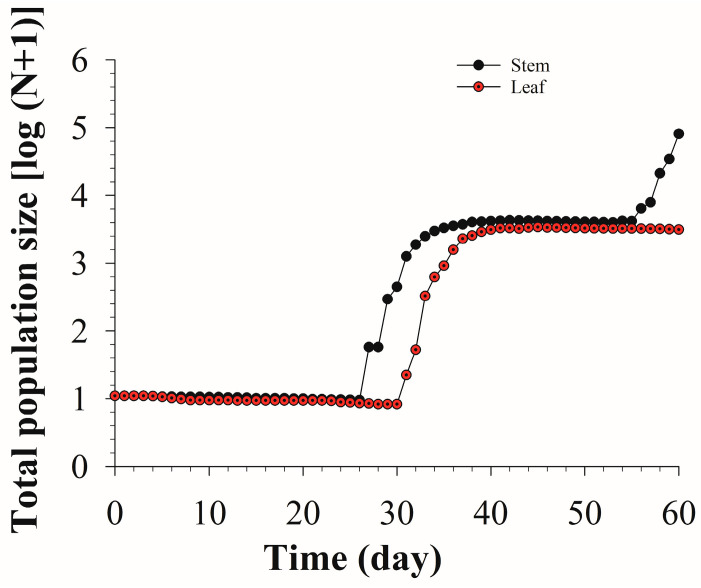
Population projection of *S. litura* fed on different tissues of asparagus.

The population dynamics of *S. litura* after feeding on different parts of asparagus for 60 days were simulated and predicted according to the data of the age-stage two-sex life table. The results showed that the minimum population size of *S. litura* fed on asparagus stems and leaves appeared at 26 d and 30 d, respectively. After that, the population began to sharply increase, and the population of *S. litura* fed on asparagus stems increased faster than that fed on asparagus leaves (Figure 5).

**Figure 6:**
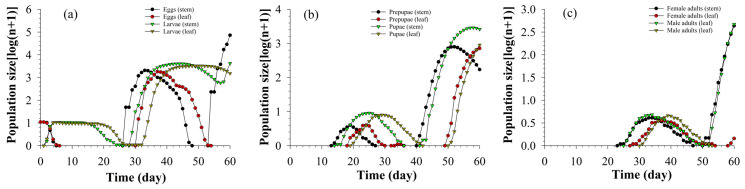
Population projection of each insect stage of eggs and larvae (**a**), prepupae and pupae (**b**), female adults and male adults (**c**) of *S. litura* fed on different tissues of asparagus.

After feeding on asparagus for 60 days, the eggs in the stems group were at the peak of the third generation, while only the second-generation eggs appeared in the leaves group. The third-generation larvae on stems were in the rising stage, while the second-generation larvae on leaves were in the declining stage. The second generation of prepupae and pupae on the stems were in the decline stage, and the prepupae and pupae on the leaves were in the second-generation peak. The male and female adults on the stems were at the peak of the second generation. The female adults on the leaves had just appeared in the second generation and were in the growth period, while the male adults on the leaves only had one generation Figure 6.

### 3.7. Feeding Preference

**Figure 7:**
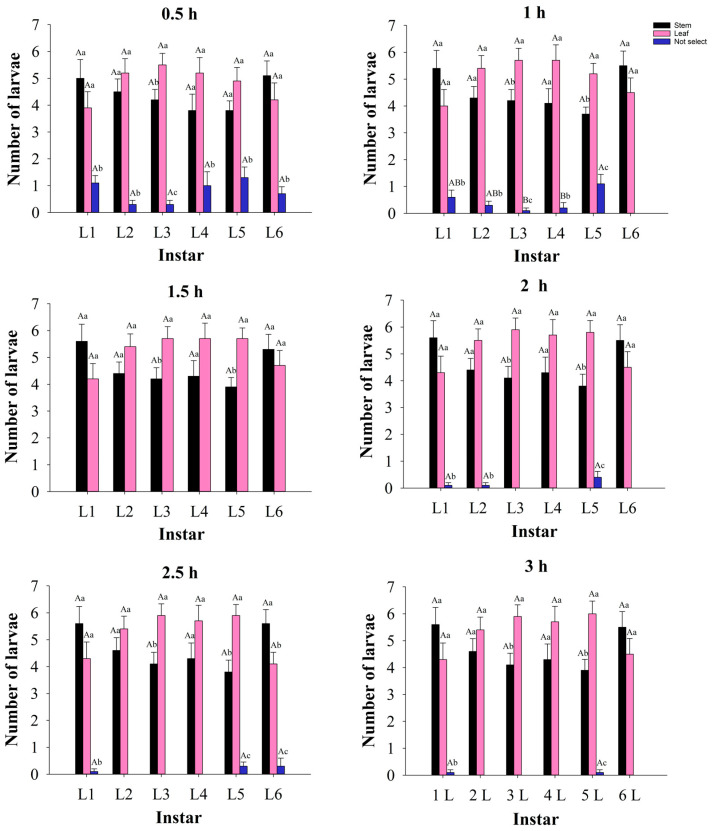
Feeding preference of each larvae stage of *S. litura* between asparagus stems and leaves. Values are mean ± SE, different lowercase letters at the same investigation time indicate that the same instar larvae are significantly different between different tissues, and different capital letters indicate that the different instar larvae are significantly different between the same tissues (*p* < 0.05).

The feeding selectivity experiment (Figure 7) showed that the feeding preference of third- and fifth-instar larvae (except 0.5 h) on stems and leaves was significantly different; they preferred feeding on leaves. The 6th instar larvae preferred to feed on stems when they were placed for 2.5 h, then on two tissues, there was no significant difference in their preference at other observation times. There was no significant difference in the host preference of the 1st, 2nd, and 4th instar larvae at any observation time. Additionally, there was no significant difference in feeding selectivity between different instar larvae to the same part of asparagus at the same time.

## 4. Discussion

As a polyphagous pest, *S. litura* has wide host adaptability, but it also shows different adaptability in different parts of the same host plant [16,56]. Our results showed that the larval, pupal, and preadult developmental durations of *S. litura* feeding on asparagus stems and leaves were significantly different. This is consistent with the results of Zhang et al. [57] on *S*. *frugiperda* and Fousséni Traore et al. [58] on *Maruca vitrata* feeding on host-different tissues. In our study, the larval, pupal, and preadult developmental duration on the leaves treatment was longer than that on the stems treatment, and the survival rate was lower than that of stem treatment. The results of Wang et al. [29] showed that the total larval duration of *S. litura* fed on asparagus leaves at 27 ± 1 °C, 70% (R. H), and 14 L:10 D was 22.7 d, which was longer than those fed on leaves (19.29 d) and stems (15.17 d) in our experiment. The developmental duration of pupae was 10.6 d, which was close to those fed on leaves (10.11 d) in our study, but longer than those fed on stems (9.42 d) in this experiment. It can be seen that the larval and pupal developmental duration of *S. litura* reared with leaves in that study and in our study was longer than those reared with stems. Different nutrients, volatile secondary substances, and epidermal characteristics among different host plants may affect the growth, development, and fecundity of insects [59,60,61]. Related studies have revealed that the levels of protein, amino acids, and sugars are high in both asparagus stems and leaves, but leaves have a lower moisture content and higher contents of flavonoids than those in asparagus stems [62]. Meanwhile, studies have shown that the water content in plants was positively correlated with the growth and development parameters of *S. litura* [63,64]. The developmental duration of insects was prolonged by flavonoids in host pants [65]. Additionally, the asparagus stems are spear-shaped with a large and thick structure, while the asparagus leaves are shaped like small needles [66]. Therefore, the development time of *S. litura* on asparagus leaves was longer than that on the stems, which may have been caused by a lower water content and higher flavonoid content in the asparagus leaves than in the asparagus stems. At the same time, small leaves may be difficult to satisfy the growth and development of larvae in a short time; this may also be one of the reasons for the significant prolongation development period of larvae on asparagus leaves. However, the specific reason needs further research and confirmation.

In any treatment, there was no significant difference in adult preoviposition period (APOP), longevity, fecundity, and oviposition days. Our results are inconsistent with the results of Tang et al. [17] and Zhang et al. [57] on *S. frugiperda* in different tissues of hosts. Their studies showed that the longevity and fecundity of fall armyworm adults on host-different tissues were significantly different. This difference may be due to the variation in the types of hosts and insects used in the experiment; however, our results are consistent with Tang et al. [67] and Moreau et al. [68]. The results of Tang et al. [67] showed that the development of *S. frugiperda* larvae feeding on shallot and onion was significantly different, but the preoviposition period, fecundity, and longevity of adults were not significantly different. There was also no significant difference in the reproductive parameters of *Lobesia botrana* in Moreau et al. [68]. This was probably because the prolonged larval development time enabled larvae to compensate for a low consumption rate or for food of poor quality and to finally reach the same longevity and fecundity as larvae that were reared on cultivars that promoted a faster larval development. So, the results of the APOP, longevity, fecundity, and oviposition days showed that there was no significant difference between the two tissues of asparagus in our study. It may also be because the larvae significantly prolonged their development time on the leaves to achieve the same fecundity as on the stems. In addition, the average number of eggs laid by a single female of *S. litura* fed on leaves was 1148.00, and the adult longevity was 9.4 days in Wang et al. [29], which was consistent with our results. The preoviposition period of adults was shorter than that of this study, and the oviposition days were longer than those of this study. That study used 70% humidity for the experiment, while we used 75% humidity for our experiment. Therefore, it may be related to asparagus varieties or the different humidity of the experimental environment.

The population parameters *r* and *λ* of *S. litura* fed on stems were 0.186 d^−1^ and 1.204 d^−1^, respectively, significantly higher than those fed on leaves (0.161 d^−1^ and 1.175 d^−1^). Then, the parameter *T* (on asparagus stems, 32.88 d) was significantly lower than that on leaves (36.88 d, *p* < 0.05). Usually, a shorter developmental duration, higher survival rate, and fecundity of insects indicate greater adaptability to the host plants [68,69]. Those results indicated that *S. litura* could complete its life cycle in both tissues, but stems were more suitable for population growth. Although there was no significant difference in the longevity and fecundity of adults fed on asparagus stems and leaves, the larval development duration, pupal development duration, and mean generation time of *S. litura* fed on asparagus stems were significantly shorter than those fed on leaves. Moreover, the survival rate, the intrinsic rate of increase, and the finite rate of increase on stems were greater than those on leaves, so the stems were more suitable for the growth and development of *S. litura* than leaves.

Feeding preference is essential for insect adaptation and survival [70]. A study by Wu [15] showed that *S. litura* larvae had different selection preferences for soybean tissues at different growth stages. Our research is consistent with this study. Our study showed that the 3rd and 5th (except for being placed for 0.5 h) instar larvae preferred to feed on leaves. The 6th instar larvae had no significant difference in the preference of stems and leaves at most observation times, except that they preferred to feed on stems at 2.5 h. There was no significant difference in the host preference of the 1st, 2nd, and 4th instar larvae at any observation time. Polyphagous insects usually prefer to feed on plants that are more suitable for their growth, development, and reproduction [52,71,72]. The present study, however, obtained the opposite result: the larvae were either non-selective or preferred to feed on asparagus leaves, whereas their fitness was higher with feeding on stems. Our results are consistent with the results of Xu et al. [73], who studied *S. frugiperda* on *Zea mays*, *Vigna unguiculata*, and *Phaseolus vulgaris*. The results showed that compared with maize, the development of *S. frugiperda* fed on cowpea leaves and kidney bean leaves was relatively slow, and the pupation rate and emergence rate were lower, indicating that maize leaves had better adaptability to the growth and development of *S. frugiperda*. However, in the larval host preference experiment, the larvae not only showed strong selectivity to the feeding host maize leaves, but also showed strong host preference for cowpea leaves and kidney bean leaves. Insects prefer to choose hosts that are conducive to their growth and development in most cases, but this is not always the case and may be related to different hosts and insect species. The physical and chemical characteristics of host plants or the status of insects will directly or indirectly affect the feeding choice of herbivorous insects [74,75,76]. The adaptation mechanism of insects to hosts is different among different insects. In the case of multiple hosts, some larvae colonize available hosts randomly and immediately. After colonization, if the host is not suitable or the number of hosts is not enough to eat, they may disperse in search of a more suitable host than the colonized host. Some insects may be affected by the volatiles of the host before making a choice on the host [77]. In this study, the 1st, 2nd, 4th, and 6th instar (except 2.5 h) larvae did not show obvious preference behavior between asparagus stems and leaves, which may be related to the high nutrient content in stems and leaves [63] or the independent selection of hosts. However, the 6th instar larvae showed obvious preference between the two hosts at 2.5 h, which may be related to the stronger activity of the older larvae. The preference of the 3rd and 5th instar (except 0.5 h) larvae to asparagus leaves may be due to their best adaptive mechanism to asparagus leaves, but we have not studied the specific adaptation mechanism, which will be our next research plan. At the same time, in the laboratory, we provided sufficient hosts and a stable experimental environment for experimental insects. The asparagus field environments are relatively complex, factors such as temperature, humidity, natural enemies, and pesticides may affect the growth and feeding preference of *S. litura*. Therefore, it is necessary to conduct more field experiments for further research. Results from this study can also provide a theoretical reference for our further research and field control. In the field, biological or chemical control methods should be used on stems and leaves to prevent *S. litura* in advance. Especially, when its number on leaves increases, it should be prevented from transferring to stems to cause more destructive damage.

## 5. Conclusions

In summary, the analysis results of life table parameters proved that *S. litura* could complete growth and development on asparagus stems or leaves; moreover, asparagus stems are more suitable for its population growth. In terms of feeding preference, at any observation time, the 3rd instar of *S. litura* preferred to consume asparagus leaves. At most observation times, the 5th instar larvae significantly preferred to feed leaves, and the 6th instar larvae had no significant difference in feeding preference between stems and leaves at most observation times. There was no significant difference in the feeding preference of 1st, 2nd, and 4th instar larvae to stems and leaves at any observation time. In the field, we can formulate more reasonable control measures to focus on the prevention and control of *S. litura* according to the preference of different insect stages to different tissues of asparagus and the inconsistent growth and development speed of *S. litura* on different tissues. It can not only improve the efficiency of pesticide use, but also reduce the amount of pesticide application, and is conducive to better control of *S. litura*.

## Figures and Tables

**Table 1:** Development duration of *S. litura* fed on different tissues of asparagus.

Developmental Stage	Stem	Leaf
Eggs	3.36 ± 0.07 a	3.46 ± 0.08 a
1st instar larvae	3.23 ± 0.07 b	3.65 ± 0.08 a
2nd instar larvae	2.39 ± 0.05 b	3.52 ± 0.09 a
3rd instar larvae	2.30 ± 0.07 b	3.17 ± 0.08 a
4th instar larvae	2.18 ± 0.08 b	3.00 ± 0.11 a
5th instar larvae	2.41 ± 0.09 b	2.85 ± 0.08 a
6th instar larvae	2.72 ± 0.10 b	3.23 ± 0.08 a
larvae	15.17 ± 0.02 b	19.29 ± 0.02 a
Prepupal	1.97 ± 0.08 a	2.21 ± 0.13 a
Pupal	9.42 ± 0.14 b	10.11 ± 0.19 a
Preadult	29.88 ± 0.29 b	35.04 ± 0.35 a

Values are mean ± SE. Different lowercase letters in the same row indicate significant differences (*p* < 0.05).

**Table 2:** Adult longevity and reproductive parameters of *S. litura* fed on different tissues of asparagus.

Biological Parameters	Stem	Leaf
Adult preoviposition period	2.52 ± 0.33 a	2.56 ± 0.33 a
Total preoviposition period	32.00 ± 0.61 b	36.56 ± 0.80 a
Fecundity	1237.92 ± 179.85 a	1168.94 ± 197.97 a
Oviposition days	3.65 ± 0.32 a	3.20 ± 0.34 a
Female longevity	9.69 ± 0.48 a	8.52 ± 0.53 a
Male longevity	9.66 ± 0.58 a	9.47 ± 0.44 a

Values are mean ± SE. Different lowercase letters in the same row indicate significant differences (*p* < 0.05).

**Table 3:** Population parameters of *S. litura* fed on different tissues of asparagus.

Population Parameters	Feeding Site	
	Stem	Leaf
Intrinsic rate of increase, *r* (d^−1^)	0.1855 ± 0.0071 a	0.1615 ± 0.0069 b
Finite rate of increase, *λ* (d^−1^)	1.2039 ± 0.085 a	1.1752 ± 0.0081 b
Net reproductive rate, *R_0_* (offspring per individual)	445.65 ± 87.74 a	385.75 ± 85.24 a
Mean generation time, *T* (d)	32.88 ± 0.58 b	36.88 ± 0.59 a

Values are mean ± SE. Different letters in the same row indicate significant differences at *p* < 0.05 level by paired bootstrap test.

## Data Availability

There is no supplementary information to reveal; all the information is contained in this manuscript.

## References

[1] Bayu M.S.Y.I., Krisnawati A. (2016). The difference growth and development of army worm (*Spodoptera litura*) on five host plants. Nusant. Biosci..

[2] Nawaz A., Ali H., Sufyan M., Dildar Gogi M., Jalal Arif M., Ali A., Qasim M., Islam W., Tayab M., Bodla I. (2019). In-vitro assessment of food consumption, utilization indices and losses promise of leafworm, *Spodoptera litura* (Fab.), on okra crop. J. Asia-Pac. Entomol..

[3] Zhang H.X., Birch J., Xie C.N., Yang H.Y., Dias G., Kong L.M., Bekhit A.E.D. (2018). Optimization of extraction parameters of antioxidant activity of extracts from New Zealand and Chinese *Asparagus officinalis* L. root cultivars. Ind. Crop. Prod..

[4] Pegiou E., Mumm R., Acharya P., de Vos R.C.H., Hall R.D. (2019). Green and white asparagus (*Asparagus officinalis*): A source of developmental, chemical and urinary intrigue. Metabolites..

[5] Chitrakar B., Zhang M., Adhikari B. (2019). Asparagus (*Asparagus officinalis*): Processing effect on nutritional and phytochemical composition of spear and hard-stem byproducts. Trends Food Sci. Technol..

[6] Chitrakar B., Zhang M., Zhang X.H., Bhandari B. (2022). Valorization of asparagus-leaf by-product through nutritionally enriched chips to evaluate the effect of powder particle size on functional properties and rutin contents. Dry. Technol..

[7] Guo H.M., Huang W.M., Tang Z.H., Ye J.A. (2017). Effect of *Asparagus officinalis* stem and leaf silage ration on productive performance, rumen fermentation parameters and blood indices of growing hu sheep. China. J. Anim. Sci..

[8] Yu E.M. (2015). The Research of Mother Fern Kept Method and Nitrogen and Potassium Fertilizer Used of Asparagus Cultured in Plastic Greenhouse. Master’s Thesis.

[9] Mao X.M., Bai C., Yang J., Ning G.Y., Liu C.C. (2021). Occurrence regularity and green control technology of Noctuidae on asparagus in greenhouse. J. Zhejiang. Agric. Sci..

[10] Zeng H.L., He L., Ye P.S., Jiang Q.P., Hua L.X., Huang L., Wei S.G., Wang M.J., Dai S.D., Zhang M. (2021). Investigation on main diseases and pests of asparagus in Sichuan province. J. Anhui. Agric. Sci..

[11] Abbas N., Shad S.A., Razaq M. (2012). Fitness cost, cross resistance and realized heritability of resistance to imidacloprid in *Spodoptera litura* (Lepidoptera: Noctuidae). Pestic. Biochem. Physiol..

[12] Night G., Gold C.S., Power A.G. (2011). Feeding behaviour and efficiency of banana weevil (*Cosmopolites sordidus*) larvae on banana cultivars of varying resistance levels. J. Appl. Entomol..

[13] Vengateswari G., Arunthirumeni M., Shivaswamy M.S., Shivakumar M.S. (2022). Effect of host plants nutrients, antioxidants, and phytochemicals on growth, development, and fecundity of *Spodoptera litura* (Fabricius) (Lepidoptera: Noctuidae). Int. J. Trop. Insect Sci..

[14] Damle M.S., Giri A.P., Sainani M.N., Gupta V.S. (2005). Higher accumulation of proteinase inhibitors in flowers than leaves and fruits as a possible basis for differential feeding preference of *Helicoverpa armigera* on tomato (*Lycopersicon esculentum* Mill, Cv. Dhanashree). Phytochemistry..

[15] Wu Q. (2021). Changes of Host Preference in *Spodoptera litura* and the Role of Plant Volatiles in Its Host Selection. Master’s Thesis.

[16] Hu Z.Z., Xu X.C., Pan L., Li M., Zeng J., Muhammad K.R., Xin G.N., Gai J.Y. (2020). Resistance analyses of soybean organs to common cutworm (*Spodoptera litura*) at different reproductive stages. Soybean Sci..

[17] Tang Q.F., Fang M., Yao L., Qiu K., Zeng Z.Y., Jin T., Li G.T. (2020). Effects of feeding different corn organizations on growth, development and nutritional indexes of *Spodoptera frugiperda*. Plant Prot..

[18] Suits R., Reisig D., Burrack H. (2017). Feeding preference and performance of *Helicoverpa zea* (Lepidoptera: Noctuidae) larvae on various soybean tissue types. Fla. Entomol..

[19] Luo N. (2021). Effect and Mechanism Analysis of Feeding on Different Parts of Wheat Seedling on the Aphid Production of *Rhopalosiphum padi*. Master’s Thesis.

[20] Tu X.Y., Chen Y.S. (2013). Feeding preference of *Brithys crini* Fabricius (Lepidoptera: Noctuidae) larvae to host plants and plant parts. North. Horticult..

[21] Tuan S.J., Yeh C.C., Atlihan R., Chi H., Tang L.C. (2016). Demography and consumption of *Spodoptera litura* (Lepidoptera: Noctuidae) reared on cabbage and taro. J. Econ. Entomol..

[22] Tuan S.J., Lee C.C., Chi H. (2014). Population and damage projection of *Spodoptera litura* (F.) on peanuts (*Arachis hypogaea* L.) under different conditions using the age-stage, two-sex life table. Pest Manag. Sci..

[23] Shahout H.A., Xu J., Yao X.M., Jia Q.D. (2011). Influence and mechanism of different host plants on the growth, development and, fecundity of reproductive system of common cutworm *Spodoptera litura* (Fabricius) (Lepidoptera: Noctuidae). Asian J. Agric. Sci..

[24] Yue W.B., Zhi J.R., Liu L., Ye M., Zhang X.Q., Zeng G. (2018). Effects of pest insect feeding and mechanical damage on the defensive enzyme activities in leaves at different parts of kidney bean plants. Acta Entomol. Sin..

[25] Jha R.K., Tuan S.J., Chi H., Tang L.C. (2014). Life table and consumption capacity of corn earworm, *Helicoverpa armigera*, fed asparagus, *Asparagus officinalis*. J Insect Sci..

[26] Duan G.Q., Zhang Z.B., Zhang H.J., Zhang Y.B., Wang J.J., Ma L.P. (2010). Preference of *lsoceras sibirica* to storage root and stem of asparagus and effects of humidity on development of its pupae. J. Environ. Entomol..

[27] Wright L.C., Cone W.W. (1988). Population statistics for the asparagus aphid, *Brachycorynella asparagi* (Homoptera: Aphididae), on different ages of asparagus foliage. Environ. Entomol..

[28] Teng H.Y., Shi P.X., Yuan Y.D., Zhang T.S., Wang D.S. (2010). Effects of feeding asparagus on development, fecundity, and survival rate of beet armyworm (*Spodoptera exigua*). J. Chang. Veg..

[29] Wang G.H., Zhao Q.G., Cui J. (2015). Effects of different foodstuff on growth and development of *Prodenia litura*. Guizhou. Agric. Sci..

[30] Zhao J., Xiao D., Zhang F., Wang S. (2015). Life tables of *Harmonia axyridis* Pallas under laboratory constant and greenhouse fluctuating temperatures. Sci. Agric. Sin..

[31] Chi H., Su H.Y. (2006). Age-stage, two-sex life tables of *Aphidius gifuensis* (Ashmead)(Hymenoptera: Braconidae) and its host *Myzus persicae* (Sulzer)(Homoptera: Aphididae) with mathematical proof of the relationship between female fecundity and the net reproductive rate. Environ. Entomol..

[32] Birch L.C. (1948). The intrinsic rate of natural increase of an insect population. J. Anim. Ecol..

[33] Chi H., Liu H. (1985). Two new methods for the study of insect population ecology. Bull. Inst. Zool., Acad. Sin..

[34] Chi H., Fu J.W., You M.S. (2019). Age-stage, two-sex life table and its application in population ecology and integrated pest management. Acta Entomol. Sin..

[35] Huang Y.B., Chi H. (2013). Life tables of *Bactrocera cucurbitae* (Diptera: Tephritidae): With an invalidation of the jackknife technique. J. Appl. Entomol..

[36] Farhadi R., Allahyari H., Chi H. (2011). Life table and predation capacity of *Hippodamia variegata* (Coleoptera: Coccinellidae) feeding on *Aphis fabae* (Hemiptera: Aphididae). Biol. Control..

[37] Chen G.M., Chi H., Wang R.C., Wang Y.P., Xu Y.Y., Li X.D., Yin P., Zheng F.Q. (2018). Demography and uncertainty of population growth of *Conogethes punctiferalis* (Lepidoptera: Crambidae) reared on five host plants with discussion on some life history statistics. J. Econ. Entomol..

[38] Dong S., Lu Z.B., Li L.L., Zhu J.S., Guan X.M., Men X.Y. (2022). Life tables of the invasive insect pest fall armyworm, *Spodoptera frugiperda* feeding on seven crops. J. Plant Prot..

[39] Liu S.T., Wang Z.Y., Liu Y.Q., Qu A.J. (2019). Life table of *Spodoptera litura* on grape. Plant Prot..

[40] Di X.Y., Yan B., Wu C.X., Yu X.F., Liu J.F., Yang M.F. (2021). Does larval rearing diet lead to premating isolation in *Spodoptera litura* (Fabricius) (Lepidoptera: Noctuidae)?. Insects..

[41] Thompson J.N. (1988). Evolutionary ecology of the relationship between oviposition preference and performance of offspring in phytophagous insects. Entomol. Exp. Appl..

[42] Fujiwara C., Takabayashi J., Yano S. (2000). Oviposition experience on a host-infested plant affects flight and antennal searching behavior of *Cotesia kariyai* toward the host-plant complex. Entomol. Exp. Appl..

[43] Sequeira R.V., McDonald J.L., Moore A.D., Wright G.A., Wright L.C. (2001). Host plant selection by *Helicoverpa* spp. in chickpea-companion cropping systems. Entomol. Exp. Appl..

[44] Zhang S.L., Liu J.F., Zou X., Yang M.F., Shang S.H. (2020). Effects of *Isaria cicadae* SLGY-2 on the development and protective enzyme activities in vivo of *Spodoptera litura*. J. Mt. Agric. Biol..

[45] Mou D.F., Lee C.C., Smith C.L., Chi H. (2015). Using viable eggs to accurately determine the demographic and predation potential of *Harmonia dimidiata* (Coleoptera: Coccinellidae). J. Appl. Entomol..

[46] Quais M.K., Ansari N.A., Wang G.Y., Zhou W.W., Zhu Z.R. (2019). Host plant salinity stress affects the development and population parameters of *Nilaparvata lugens* (Hemiptera: Delphacidae). Environ. Entomol..

[47] Chi H. (1988). Life-table analysis incorporating both sexes and variable development rates among individuals. Environ. Entomol..

[48] Huang H.W., Chi H., Simth C.L. (2017). Linking demography and consumption of *Henosepilachna vigintioctopunctata* (Coleoptera: Coccinellidae) fed on *Solanum photeinocarpum* (Solanales:Solanaceae): With a new method to project the uncertainty of population growth and consumption. Environ. Entomol..

[49] He L., Ge S., Zhang H., He W., Yan R., Wu K. (2021). Photoregime affects development, reproduction, and flight performance of the invasive fall armyworm (Lepidoptera: Noctuidae) in China. Environ. Entomol..

[50] Del Pino M., Cabello T., Hernández-Suárez E. (2020). Age-stage, two-sex life table of *Chrysodeixis chalcites* (Lepidoptera: Noctuidae) at constant temperatures on semi-synthetic diet. Environ. Entomol..

[51] Chi H. (2021). TIMING-MSChart: A Computer Program for the Population Projection Based on Age-Stage, Two-Sex Life Table.

[52] Huang Q., Qin J.M., Huang X.F., Zhong Y., Li C., Wu B.Q., Huang S.S., Huang F.K., Ling Y., Long L.P. (2022). Feeding preference and growth adaptability of *Spodoptera frugiperda* on different sugarcane varieties and *Rottboellia cochinchinensis*. J. South China Agric. Univ..

[53] He L., Wu Q., Gao X., Wu K. (2021). Population life tables for the invasive fall armyworm, *Spodoptera frugiperda* fed on major oil crops planted in China. J. Integr. Agric..

[54] Chi H. (2021). TWOSEX-MSChart: A Computer Program for The Age-Stage, Two-Sex Life Table Analysis.

[55] Bahari F., Fathipour Y., Talebi A.A., Alipour Z. (2018). Long-term feeding on greenhouse cucumber affects life table parameters of two-spotted spider mite and its predator *Phytoseiulus persimilis*. Syst. Appl. Acarol..

[56] Wan P., Wu K.M., Huang M.S., Yu D.Z., Wu J.P. (2008). Population Dynamics of *Spodoptera litura* (Lepidoptera: Noctuidae) on Bt Cotton in the Yangtze River Valley of China. Environ. Entomol..

[57] Zhang Z., Jiang Y., Li X., Zhang A., Zhu X., Zhang Y. (2021). Effects of different wheat tissues on the population parameters of the fall armyworm (*Spodoptera frugiperda*). Agronomy.

[58] Traore F., Dabire-Binso C.L., Ba N.M., Sanon A., Pittendrigh B.R. (2013). Feeding preferences of the legume pod borer *Maruca vitrata* (Lepidoptera: Crambidae) larvae and suitability of different flower parts for larval development. Int. J. Trop. Insect Sci..

[59] Yuan E.L., Yan H.Y., Gao J., Guo H.J., Ge F., Sun Y.C. (2019). Increases in genistein in medicago sativa confer resistance against the *Pisum* host race of *Acyrthosiphon pisum*. Insects.

[60] Chun H.Q., Li J.H., Chen Y.D., Chen T., Zhao Y.G., Li Y.H. (2011). Effects of host plants and their secondary substances on *Spodoptera litura* (Lepidoptera: Noctuidae). Hubei. Agric. Sci..

[61] Duan Y.J., He X., He H.J., Liu W. (2020). Effects of physical characteristics of *Rhododendron* leaves on hostselection of *Stephanitis pyriodes* Scott. Southwest Chin. J. Agric. Sci..

[62] Guan Y.J., Zhou L.Y., Bi J.F., Yi J.Y., Li S.R. (2015). Evaluation of nutritive composition and antioxidant activity in different parts of green asparagus. Sci. Technol. Technol. Food Ind..

[63] Subramanian K., Raja N., Ignacimuthu S. (2006). Feeding performance of *Spodoptera litura* on different cotton cultivars. J. Entomol. Res..

[64] Tuan S.J., Li N.J., Yeh C.C. (2015). Growth performance and biometric characteristics of *Spodoptera litura* (Lepidoptera: Noctuidae) reared on different host plants. J. Econ. Entomol..

[65] Wang M., Jiang D., Meng Z.J., Yan S.C. (2020). Adaptability of larval growth and development in *Hyphantria cunea* to different host plant secondary metabolites. J. Northeast For. Univ..

[66] Bai Y.Y., Kelly J.F. (1999). A study of photosynthetic activities of eight asparagus genotypes under field conditions. J. Amer. Soc. Hort. Sci..

[67] Tang Y., Guo J.F., Wang Q.Y., Tai H.K., He K.L., Wang Z.Y. (2020). Potential threat posed by the fall armyworm *Spodoptera frugiperda* to shallot and onion crops. Chin. J. Appl. Entomol..

[68] Moreau J., Benrey B., Thiéry D. (2006). Grape variety affects larval performance and also female reproductive performance of the European grapevine moth *Lobesia botrana* (Lepidoptera: Tortricidae). Bull. Entomol. Res..

[69] Li D.W., Qin Z.Q., Lou Y.W., Song X.P., Wei C.Y., Ding H.Z., Lin C. (2018). Effects of *Zizania latifolia* and sweet corn on growth and development of *Chilo infuscatellus* Snellen. J. South. Agric..

[70] Zhang Z.J., Zhang S.S., Niu B.L., Ji D.F., Liu X.J., Li M.W., Bai H., Palli S.R., Wang C.Z., Tan A.J. (2019). A determining factor for insect feeding preference in the silkworm, *Bombyx mori*. PLoS Biol..

[71] Rajapakse C.N.K., Walter G.H. (2007). Polyphagy and primary host plants: Oviposition preference versus larval performance in the lepidopteran pest *Helicoverpa armigera*. Arthropod Plant Interact..

[72] Liu Z., Scheirs J., Heckel D.G. (2012). Trade-offs of host use between generalist and specialist *Helicoverpa sibling* species: Adult oviposition and larval performance. Oecologia.

[73] Xu D., Li W.J., Wang L., Yin H.C., Cong S.B., Yang N.N., Xie Y.L., Wan P. (2022). Feeding and oviposition preference and adaptability of the fall armyworm, *Spodoptera frugiperda* (Lepidoptera Noctuidae), on two leguminous vegetables. J. Environ. Entomol..

[74] Wang Y.H., Chen W.H., Yi C.H., Xiao W.X., Duan X.A., Liu Q., Zhang X.M. (2022). Oviposition and feeding selection of fall armyworm on four maize varieties. Jiangsu Agric. Sci..

[75] Zhao R., Niu Y., Wang Y.L., Wang M., Zhao M.M., Zhang X.C., Jiao L.Y.L., Han Y.H., Li R. (2022). Effects of physical characteristics and nutrients of host plants on the host selectivity of *Pyrrhocoris tibialis* (Hemiptera: Pyrrhocoridae). Acta Entomologica Sinica..

[76] Yuan G.G., Zhao L.C., Du Y.W., Yu H., Shi X.B., Chen W.C., Chen G. (2022). Repellence or attraction: Secondary metabolites in pepper mediate attraction and defense against *Spodoptera litura*. Pest Manag. Sci..

[77] Wijerathna D.M.I.J., Ranaweera P.H., Perera R.N.N., Dissanayake M.L.M.C., Kumara J.B.D.A.P. (2021). Biology and feeding preferences of *Spodoptera Frugiperda* (Lepidoptera: Noctuidae) on maize and selected vegetable crops. J. Agric. Sci. Sri Lanka..

